# Significant Association of KIR2DL3-HLA-C1 Combination with Cerebral Malaria and Implications for Co-evolution of KIR and HLA

**DOI:** 10.1371/journal.ppat.1002565

**Published:** 2012-03-08

**Authors:** Kouyuki Hirayasu, Jun Ohashi, Koichi Kashiwase, Hathairad Hananantachai, Izumi Naka, Atsuko Ogawa, Minoko Takanashi, Masahiro Satake, Kazunori Nakajima, Peter Parham, Hisashi Arase, Katsushi Tokunaga, Jintana Patarapotikul, Toshio Yabe

**Affiliations:** 1 Department of Human Genetics, Graduate School of Medicine, The University of Tokyo, Bunkyo-ku, Tokyo, Japan; 2 Japan Society for the Promotion of Science, Tokyo, Japan; 3 Japanese Red Cross Tokyo Blood Center, Koto-ku, Tokyo, Japan; 4 Department of Immunochemistry, WPI Immunology Frontier Research Center, Osaka University, Suita, Osaka, Japan; 5 Doctoral Program in Life System Medical Sciences, Graduate School of Comprehensive Human Sciences, University of Tsukuba, Tsukuba, Ibaraki, Japan; 6 Faculty of Tropical Medicine, Mahidol University, Bangkok, Thailand; 7 Department of Microbiology and Immunology, School of Medicine, Stanford University, Stanford, California, United States of America; 8 Department of Immunochemistry, Research Institute for Microbial Diseases, Osaka University, Suita, Osaka, Japan; 9 Core Research for Evolutional Science and Technology, Japan Science and Technology Agency, Saitama, Japan; Case Western Reserve University, United States of America

## Abstract

Cerebral malaria is a major, life-threatening complication of *Plasmodium falciparum* malaria, and has very high mortality rate. In murine malaria models, natural killer (NK) cell responses have been shown to play a crucial role in the pathogenesis of cerebral malaria. To investigate the role of NK cells in the developmental process of human cerebral malaria, we conducted a case-control study examining genotypes for killer immunoglobulin-like receptors (KIR) and their human leukocyte antigen (HLA) class I ligands in 477 malaria patients. We found that the combination of KIR2DL3 and its cognate HLA-C1 ligand was significantly associated with the development of cerebral malaria when compared with non-cerebral malaria (odds ratio 3.14, 95% confidence interval 1.52–6.48, *P* = 0.00079, corrected P = 0.02). In contrast, no other KIR-HLA pairs showed a significant association with cerebral malaria, suggesting that the NK cell repertoire shaped by the KIR2DL3-HLA-C1 interaction shows certain functional responses that facilitate development of cerebral malaria. Furthermore, the frequency of the KIR2DL3-HLA-C1 combination was found to be significantly lower in malaria high-endemic populations. These results suggest that natural selection has reduced the frequency of the KIR2DL3-HLA-C1 combination in malaria high-endemic populations because of the propensity of interaction between KIR2DL3 and C1 to favor development of cerebral malaria. Our findings provide one possible explanation for KIR-HLA co-evolution driven by a microbial pathogen, and its effect on the global distribution of malaria, KIR and HLA.

## Author Summary

NK cells play an important role in early defense against pathogens. Killer immunoglobulin-like receptors (KIR) are a diverse family of activating and inhibitory receptors expressed on human NK cells. Some inhibitory KIRs recognize human leukocyte antigen (HLA) class I molecules as their ligands. The *KIR* loci exhibit presence or absence polymorphism, and thus, some individuals lack particular KIR-HLA receptor-ligand pairs, which affects their NK cell responses. We herein show that presence of both KIR2DL3 and its cognate HLA-C1 ligand in malaria patients was strongly associated with the development of human cerebral malaria. This result suggests that NK cells from the patients carrying both KIR2DL3 and HLA-C1 exhibit functional responses that facilitate development of cerebral malaria. In addition, the gene frequency of the KIR2DL3 and HLA-C1 combination was found to be significantly lower in populations with high-endemic malaria. These observations suggest that the combination of KIR2DL3 and HLA-C1 has decreased in malaria high-endemic populations under selection from cerebral malaria, a major life-threatening complication of *Plasmodium falciparum* malaria.

## Introduction

Malaria is a serious infectious disease, affecting over 300 million people and causing more than 1 million deaths annually worldwide [1]. Cerebral malaria is a major, life-threatening complication of *Plasmodium falciparum* malaria, and has a high mortality rate [2]. Host immune system and genetic factors have been considered to play a crucial role in the pathogenesis of cerebral malaria. In experimental models, the polymorphic loci responsible for susceptibility to cerebral malaria were shown to map to the natural killer (NK) complex region on mouse chromosome 6, which contains clustered genes encoding NK cell receptors [3]. Furthermore, NK cell depletion resulted in significant protection against cerebral malaria, suggesting the involvement of NK activity in its pathogenesis [4]. In humans, NK cells are an early source of IFN-γ in response to malaria infection [5], [6], and this cytokine is known to be potentially involved in the pathogenesis of cerebral malaria [7]–[9]. NK cells also show differences in responsiveness to *P. falciparum*-infected erythrocytes among malaria-naive donors [6], [10], [11], suggesting the presence of a genetic determinant for heterogeneous NK responsiveness. These observations have suggested that the genes encoding NK receptors and their ligands have critical roles in the development of cerebral malaria in humans.

Killer immunoglobulin-like receptors (KIR) are a diverse family of activating and inhibitory receptors expressed on human NK cells, and a subset of T cells. Seventeen different *KIR* genes have been identified to date [12]. The *KIR* loci exhibit high levels of genetic polymorphism in terms of gene content (e.g., presence or absence of a gene) and allelic diversity, which are considered to have been shaped by natural selection [13]–[15]. Some inhibitory KIRs recognize human leukocyte antigen (HLA) class I molecules as their ligands. KIR2DL1 recognizes HLA-C group 2 (HLA-C2) allotypes having asparagine at amino acid position 80, whereas KIR2DL2 and KIR2DL3 recognize HLA-C group 1 (HLA-C1) allotypes having lysine at amino acid position 80 [16]. KIR2DL2 and KIR2DL3 also recognize HLA-B*4601, which acquired the C1 epitope by gene conversion [17]. KIR3DL1 recognizes HLA-A and HLA-B allotypes having the Bw4 epitope determined by amino acid positions 77–83 [18], [19]. Because both *HLA* and *KIR* genes are located on different chromosomes and segregate independently, some individuals lack particular KIR-HLA receptor-ligand pairs. Numerous studies have shown that certain KIR-HLA receptor-ligand combinations are associated with susceptibility to infectious and autoimmune diseases, such as clearance of HCV, microscopic polyangiitis, type 1 diabetes and HIV disease progression [20]–[23].

Based on the above observations, we hypothesized that KIR-HLA receptor-ligand combinations are associated with cerebral malaria, and KIR-HLA receptor-ligand diversity has been shaped by fatal malaria as a selective pressure in malaria-high endemic regions. To test this hypothesis, we first examined the possible association between KIR-HLA receptor-ligand combinations and cerebral malaria in Thailand. We show herein that the KIR2DL3-HLAC1 receptor-ligand pair is significantly associated with the development of cerebral malaria. In addition, comparison of both the *KIR2DL3* and *HLA-C1* gene frequencies between malaria high-endemic and low-endemic populations suggest that natural selection has acted on both *KIR2DL3* and *HLA-C1*. To our knowledge, this is the first genetic association study to suggest an influence of NK cells in the pathogenesis of cerebral malaria on *KIR* and *HLA* frequencies in human populations where malaria is endemic.

## Results

**Figure 1:**
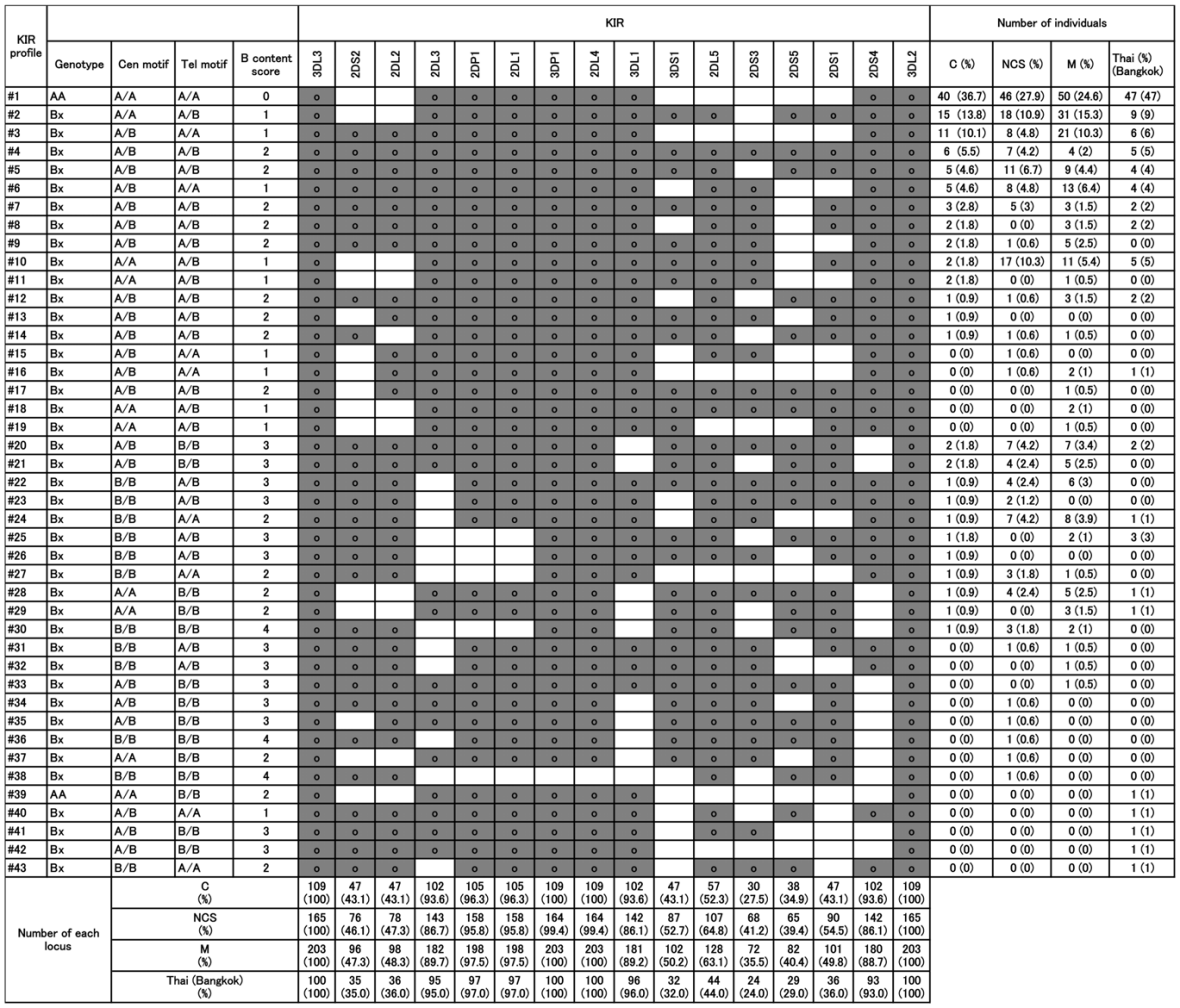
*KIR* gene profiles in our study populations. *KIR* genotyping was performed in 203 mild malaria (M), 165 non-cerebral severe malaria (NCS) and 109 cerebral malaria (C) patients in Thailand. A total of 38 *KIR* gene profiles were identified. The presence of *KIR* genes is indicated by grey shading. *KIR* gene profiles of the Thai population in Bangkok available from the Allele Frequency Net Database (population: Thailand Bangkok KIR pop 2) are shown.

To test our hypothesis that shaping of KIR-HLA receptor-ligand diversity in human populations has been affected by cerebral malaria, a life-threatening complication of malaria, we first searched for specific KIR-HLA receptor-ligand combinations associated with cerebral malaria. To this end, we analyzed 477 malaria patients living in Northwest Thailand. Because other ethnic groups such as Karen and Burmese also resided in this area, we recruited only those patients who self-identified as Thai, excluding others from the analyses. Since the primary interest of this study was the development of cerebral malaria after infection, we selected mild (n = 203) and non-cerebral severe (n = 165) malaria patient groups as controls to compare with the study group of cerebral malaria patients (n = 109). Figure 1 and Table 1 show the *KIR* genotypes and the frequency of KIR-HLA receptor-ligand combinations in the three groups. Among the six inhibitory KIR-HLA receptor-ligand pairs, the KIR2DL3-HLA-C1 pair was significantly more frequent in the cerebral malaria group when compared with non-cerebral severe (odds ratio (OR) 3.44, 95% confidence interval (95%CI) 1.59–7.43, *P* = 0.0010, corrected P (*P*c) = 0.03) and mild malaria (OR 2.90, 95%CI 1.35–6.21, *P* = 0.004, *P*c = 0.12) groups. In contrast, other KIR-HLA pairs showed no significant associations with cerebral malaria. When non-cerebral severe and mild malaria were merged into one group as non-cerebral malaria, the most significant association was obtained with the combination of KIR2DL3 and HLA-C1 and cerebral malaria, as compared to the non-cerebral malaria group (OR 3.14, 95%CI 1.52–6.48, *P* = 0.00079, *P*c = 0.02). For comparison, we also examined the HLA-C1, C2 and Bw4 frequencies within our Thai malaria patient groups with those of other populations in Thailand that were HLA typed to four digit resolution, and obtained from the Allele Frequency Net Database (population: Thailand) [24]. Although there were no significant differences in the frequencies of HLA-C1, C2, Bw4 and each individual HLA-C allele between these populations (Table 2 and supplementary table S1), the genotype frequencies of HLA-C1 and C2 showed statistically significant difference between cerebral and non-cerebral severe malaria (*P* = 0.008), and between cerebral and mild malaria groups (*P* = 0.002). These associations are presumably due to HLA-C1, because the carrier frequencies of HLA-C1 were significantly higher in the cerebral malaria group, compared with the non-cerebral malaria group (OR 7.08, 95%CI 1.69–29.7, *P* = 0.001, Table 2), but those of HLA-C2 were not significantly low (Table 2). In addition, a significant association of the HLA-C1 positivity with cerebral malaria might be secondary resulting from the combinatory effect of KIR2DL3-HLA-C1, because the combination of KIR2DL3 and HLA-C1 was more significantly associated with cerebral malaria than HLA-C1 alone (*P* = 0.00079 vs. *P* = 0.001). This is also supported by the observations that HLA-C1 in combination with KIR2DL2, another HLA-C1 receptor, showed no significant association with cerebral malaria, and in HLA-C1 positive individuals, KIR2DL3 positivity showed a trend for association with cerebral malaria (Table 2), although this failed to reach statistical significance (OR = 1.89, 95%CI = 0.82–4.37, *P* = 0.15). In this regard, however, the result should be interpreted with caution because we could not evaluate the independent effect of HLA-C1 alone and in combination with KIR2DL3 by logistic regression analysis (data not shown), because most cerebral malaria patients (100 of 109 patients) had both HLA-C1 and KIR2DL3. Therefore, we cannot rule out the possibility of the independent effect of HLA-C1 on the development of cerebral malaria. To further investigate potentially functional combinations of KIR2DL3 and each HLA-C1 allele, we compared the frequencies of the combinations of KIR2DL3 and each HLA-C1 allele (C*01, C*03, C*07, C*08, C*12, and C*14) between malaria patient groups. However, there were no significant differences (supplementary table S2), suggesting that the present association does not come from a specific HLA-C1 allele.

**Table 1:** Frequencies of KIR-HLA receptor-ligand pairs in malaria patient groups.

KIR-HLA receptor-ligand pair	Cerebral (n = 109)	Non-cerebral severe (n = 165)	Mild (n = 203)
KIR2DL1-HLA-C2	0.367	0.485	0.379
KIR2DL2-HLA-C1	0.431	0.412	0.419
KIR2DL2-B*4601	0.128	0.067	0.103
KIR2DL3-HLA-C1^a^	0.917	0.764	0.793
KIR2DL3-B*4601	0.229	0.109	0.158
KIR3DL1-HLA-Bw4^b^	0.780	0.715	0.690
KIR2DS1-HLA-C2^c^	0.174	0.321	0.212
KIR2DS4-HLA-C^d^	0.385	0.303	0.320
KIR2DS4-HLA-A11	0.440	0.479	0.399
KIR3DS1-HLA-Bw4^b^	0.358	0.442	0.389

a OR (95%CI) = 3.44 (1.59–7.43), *P* = 0.001, *Pc* = 0.03(Cerebral vs. Non-cerebral severe).

OR (95%CI) = 2.90 (1.35–6.21), *P* = 0.004, *Pc* = 0.12 (Cerebral vs. Mild).

b HLA-Bw4 epitope of either HLA-A or HLA-B, or both.

c OR (95%CI) = 2.24 (1.24–4.06), *P* = 0.008, *Pc* = 0.24 (Non-cerebral severe vs. Cerebral), OR (95%CI) = 1.76 (1.10–2.81), P = 0.02, *Pc* = 0.69 (Non-cerebral severe vs. Mild).

d Putative HLA-C ligands for KIR2DS4 include C*16:01, C*01:02, C*14:02, C*05:01, C*02:02, and C*04:01.

**Table 2:** Frequencies of the HLA-C1, C2 and Bw4 in malaria patient groups and the Thai population.

	Cerebral	Non-cerebral severe	Mild	Thailand^f^
Allele				
Bw4	0.404	0.415	0.399	0.409
C1	0.798	0.700	0.741	0.785
C2	0.202	0.300	0.259	0.216
Genotype^a^				
C1/C1	0.615	0.509	0.606	N/A
C1/C2	0.367	0.382	0.271	N/A
C2/C2	0.018	0.109	0.123	N/A
C1 Carrier^d^				
C1+	0.982	0.891^b^	0.877^c^	N/A
C1−	0.018	0.109	0.123	N/A
C2 Carrier				
C2+	0.385	0.491	0.394	N/A
C2−	0.615	0.509	0.606	N/A
C1+^e^				
2DL2/2DL2	0.065	0.143	0.096	N/A
2DL2/2DL3	0.374	0.320	0.382	N/A
2DL3/2DL3	0.561	0.537	0.522	N/A

a *P* = 0.008 (Cerebral vs non-cerebral severe malaria, *Pc* = 0.41), *P* = 0.002 (Cerebral vs mild malaria, *Pc* = 0.10).

b OR (95%CI) = 7.51 (1.74–32.4), *P* = 0.004 (Cerebral vs non-cerebral severe malaria, *Pc* = 0.20).

c OR (95%CI) = 6.55 (1.49–28.8), *P* = 0.001 (Cerebral vs mild malaria, *Pc* = 0.05).

d OR (95%CI) = 7.08 (1.69–29.7), *P* = 0.001 (Cerebral vs non-cerebral malaria, *Pc* = 0.05).

e OR (95%CI) = 1.89 (0.82–4.37), *P* = 0.15 for 2DL3 positivity (Cerebral vs non-cerebral malaria).

f HLA-C allele frequencies in Thailand are available from the Allele Frequency Net Database (population: Thailand).

N/A: not available.

Some activating KIRs are also reported to bind to particular HLA class I molecules, although some of these claims are controversial. The combination of KIR3DS1 and HLA-Bw4 is associated with slower progression of HIV infection [25], and under some condition, KIR3DS1 is thought to recognize HLA-Bw4. KIR2DS1 recognizes HLA-C2, depending on the presented peptide [26]. KIR2DS4 binds to HLA-A11, and some HLA-C alleles (C*16:01, C*01:02, C*14:02, C*05:01, C*02:02, and C*04:01) [27]. When we examined the possible association of these combinations between three malaria patient groups, we observed no significant association except for KIR2DS1-HLA-C2, which was significantly associated with non-cerebral severe malaria (Table 1).

Since strong linkage disequilibrium (LD) is a prominent feature of *KIR* region, *KIR* gene profiles were classified based on the centromeric and telomeric regions of the *KIR* A and B haplotypes (Cen-A/B and Tel-A/B) as described previously (Figure 1) [28]–[30]. When we compared the Cen-A/B and Tel-A/B frequencies between our malaria patient groups, there were no significant differences (Table 3). Since *KIR2DL3* is on the centromeric *KIR* A haplotype, the presence of both centromeric *KIR* A haplotype and HLA-C1 was also significantly associated with cerebral malaria when compared with non-cerebral malaria (OR 3.14, 95%CI 1.52–6.48, *P* = 0.00079). Because the presence or absence of KIR2DL1 is a simple distinction of two centromeric *KIR* B regions and *KIR2DL1* is in positive linkage disequilibrium with *KIR2DL3*, we also examined whether the presence or absence of KIR2DL1 in combination with HLA-C1 was associated with cerebral malaria. However, this did not reach statistical significance (*P*>0.01). We also compared our malaria patient groups with a Thai population from Bangkok, which was genotyped for all of the *KIR* loci, and available from the Allele Frequency Net Database (population: Thailand Bangkok KIR pop 2) [24]. *KIR* genotype frequencies showed significant difference between the Thai population (Bangkok) and malaria patient groups (Table 3). Because there is no information available about exposure to *P. falciparum* in these data on Bangkok Thais, we cannot distinguish at present whether the significant difference between the Bangkok population and our malaria patient groups results from different genetic backgrounds or susceptibility to infection. These observations suggest that KIR2DL3 in combination with HLA-C1 is primarily associated with the development of cerebral malaria. For comparison, we also analyzed *KIR* carrier, profiles, the *KIR* AA and Bx genotype frequencies, between the pairs of malaria patient groups in this study. However, none of them showed significant differences (Figure 1 and Table 3).

**Table 3:** Frequencies of centromeric and telomeric *KIR* genotypes in malaria patient groups and the Thai population.

	Cerebral (n = 109)	Non-cerebral severe (n = 165)	Mild (n = 203)	Thai (Bangkok)^b^ (n = 100)
Genotype^a^				
AA	0.367	0.279	0.246	0.480
Bx	0.633	0.721	0.754	0.520
Cen motif				
AA	0.560	0.521	0.512	0.640
AB	0.376	0.345	0.384	0.310
BB	0.064	0.133	0.103	0.050
Tel motif				
AA	0.532	0.448	0.468	0.610
AB	0.404	0.412	0.419	0.320
BB	0.064	0.139	0.113	0.070

a *P* = 0.12 (Cerebral vs. Thai), *P* = 0.001 (Non-cerebral severe vs. Thai), *P*<0.001 (Mild vs. Thai).

b KIR data in Bangkok was obtained from the Allele Frequency Net Database (population: Thailand Bangkok KIR pop 2).

In order to rule out the possibility of spurious associations resulting from population stratification, association analyses between cerebral and non-cerebral malaria were applied to random combinations of two single nucleotide polymorphisms (SNPs), which exhibit no LD and are independent of KIR and HLA. To this end, we used a total of 18 SNPs, which were previously genotyped [31]–[36], or additionally genotyped in this study as candidate SNPs for susceptibility to cerebral malaria. Those 18 SNPs were divided into 11 neutral and 7 non-neutral SNPs, as evaluated by heterozygosity, *F*_ST_ and iHS statistics using the Human Evolution Database (supplementary table S3 and supplementary table S4) [37]. We defined the SNPs as non-neutral when one of the three statistics reached statistical significance. We analyzed neutral and non-neutral SNPs separately. A total of 220 and 84 random combinations of two SNPs from neutral and non-neutral SNPs were obtained, respectively. When we performed association analyses using those SNPs on cerebral and non-cerebral malaria patients, neither distribution of p values was biased toward false positive association (supplementary Figure S1, P>0.01, df = 19), indicating that cerebral and non-cerebral malaria groups have no significant population structure. Therefore, these data suggest that the significant association of the KIR2DL3-HLA-C1 combination with cerebral malaria observed in this study is not due to the population stratification.

Malaria is one of the strongest selective pressures acting on the human genome. This is evident from the similarity in the global distributions of endemic malaria and the red blood cell disorders that confer protection against malaria [38]. Thus variants providing resistance to malaria were driven to high frequency in malaria-endemic areas. As cerebral malaria is a life-threatening disease and a potential selective force, it is possible to detect the signature of natural selection acting on the genes associated with cerebral malaria by comparing the allele frequencies of genes between malaria high-endemic and low-endemic populations. Thus, we tested the hypothesis that the frequency of the KIR2DL3-HLA-C1 combination is lower in malaria high-endemic populations due to natural selection by fatal malaria. To this end, a total of 29 worldwide populations, for which both *KIR2DL3* and *HLA-C1* gene frequencies were available from an earlier study [15], were analyzed.

**Figure 2:**
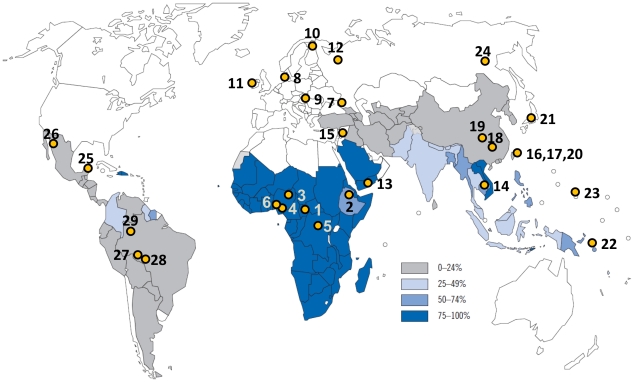
Geographical distribution of *P. falciparum* malaria cases, and the location of the 29 populations. Data for the 29 populations were obtained from an earlier report (Single et al., 2007). Plotted numbers correspond to the populations in Table 4.

Table 4 and Figure 2 show the gene frequencies of *KIR* and *HLA*, and the location of the 29 populations plotted on a world map of estimated percentage of malaria cases due to *P. falciparum*, respectively. We used the product of *KIR2DL3* and *HLA-C1* gene frequencies in a population as a measure, which we call the GF*GF index, of the frequency of KIR2DL3-HLA-C1 combination in that population.

**Table 4:** Gene frequencies of *KIR* and *HLA* in 29 worldwide populations.

					Gene frequency						
No. of location^a^	Population	Continent	Malaria endemicity	n	C1	C2	Bw4	2DL1	2DL2	2DL3	3DL1
1	Biaka	Africa	high	69	0.48	0.52	0.54	0.83	0.39	0.61	0.99
2	Ethiopian	Africa	high	31	0.48	0.52	0.55	0.69	0.52	0.48	0.87
3	Hausa	Africa	high	37	0.39	0.61	0.54	1.00	0.20	0.80	0.98
4	Ibo	Africa	high	48	0.40	0.60	0.53	0.79	0.42	0.58	0.97
5	Mbuti	Africa	high	38	0.49	0.51	0.48	0.76	0.52	0.48	0.93
6	Yoruba	Africa	high	75	0.41	0.59	0.41	1.00	0.23	0.77	0.93
7	Adygei	Europe	low	54	0.46	0.54	0.53	1.00	0.29	0.71	0.81
8	Danish	Europe	low	50	0.61	0.39	0.29	0.86	0.28	0.72	0.80
9	European	Europe	low	91	0.58	0.42	0.34	0.85	0.27	0.73	0.77
10	Finns	Europe	low	35	0.57	0.43	0.30	1.00	0.21	0.79	0.69
11	Irish	Europe	low	94	0.65	0.35	0.41	0.90	0.30	0.70	0.82
12	Russian	Europe	low	47	0.62	0.38	0.43	0.75	0.29	0.71	0.88
13	Yemenites	Asia	high	43	0.52	0.48	0.44	0.85	0.26	0.74	0.81
14	Cambodian	Asia	high	22	0.64	0.36	0.44	0.70	0.30	0.70	0.74
15	Druze	Asia	low	116	0.47	0.53	0.44	0.81	0.47	0.53	0.83
16	Ami	Asia	low	40	0.83	0.18	0.03	0.72	0.39	0.61	0.84
17	Atayal	Asia	low	42	0.95	0.05	0.00	1.00	0.00	1.00	0.83
18	Hakka	Asia	low	40	0.85	0.15	0.33	1.00	0.18	0.82	0.73
19	Han_SF	Asia	low	59	0.81	0.19	0.44	1.00	0.07	0.93	0.81
20	Han_Taiwan	Asia	low	48	0.81	0.19	0.54	0.86	0.13	0.87	0.80
21	Japan	Asia	low	49	0.89	0.11	0.36	1.00	0.04	0.96	0.73
22	Nasioi	Australia/Oceania	high	22	0.86	0.14	0.13	0.70	0.68	0.32	0.43
23	Micronesia	Australia/Oceania	low	36	0.32	0.68	0.04	0.83	0.23	0.77	0.73
24	Yakut	Asia	low	51	0.58	0.42	0.65	0.86	0.18	0.82	0.77
25	Maya	North America	low	50	0.76	0.24	0.04	0.76	0.22	0.78	0.69
26	Pima	North America	low	99	0.67	0.34	0.12	0.65	0.38	0.62	0.53
27	Karitiana	South America	low	55	0.64	0.36	0.00	0.62	0.41	0.59	0.51
28	Surui	South America	low	46	0.68	0.32	0.04	0.85	0.14	0.86	0.80
29	Ticuna	South America	low	65	0.69	0.31	0.17	0.75	0.23	0.77	0.74

a No. of locations is plotted in Figure 2. Data for the 29 populations were obtained from an earlier report (Single et al., 2007) [15].

**Figure 3:**
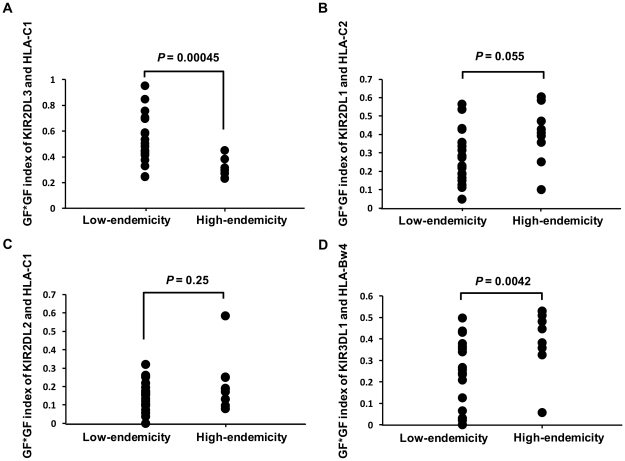
Comparison of GF*GF indices for receptor-ligand pairs between *P. falciparum* malaria high- and low-endemic populations. (A) KIR2DL3-HLA-C1, (B) KIR2DL1-HLA-C2, (C) KIR2DL2-HLA-C1 and (D) KIR3DL1-HLA-Bw4 receptor-ligand pairs were analysed. GF indicates gene frequency. The GF*GF index is defined as the product of two different gene frequencies. Vertical and horizontal axes indicate the GF*GF index of KIR-HLA receptor-ligand pair, and malaria endemicity for the 29 populations, respectively. P values were calculated using the Wilcoxon rank sum test. Data for the 29 populations were obtained from an earlier report (Single et al., 2007) [15].

**Figure 4:**
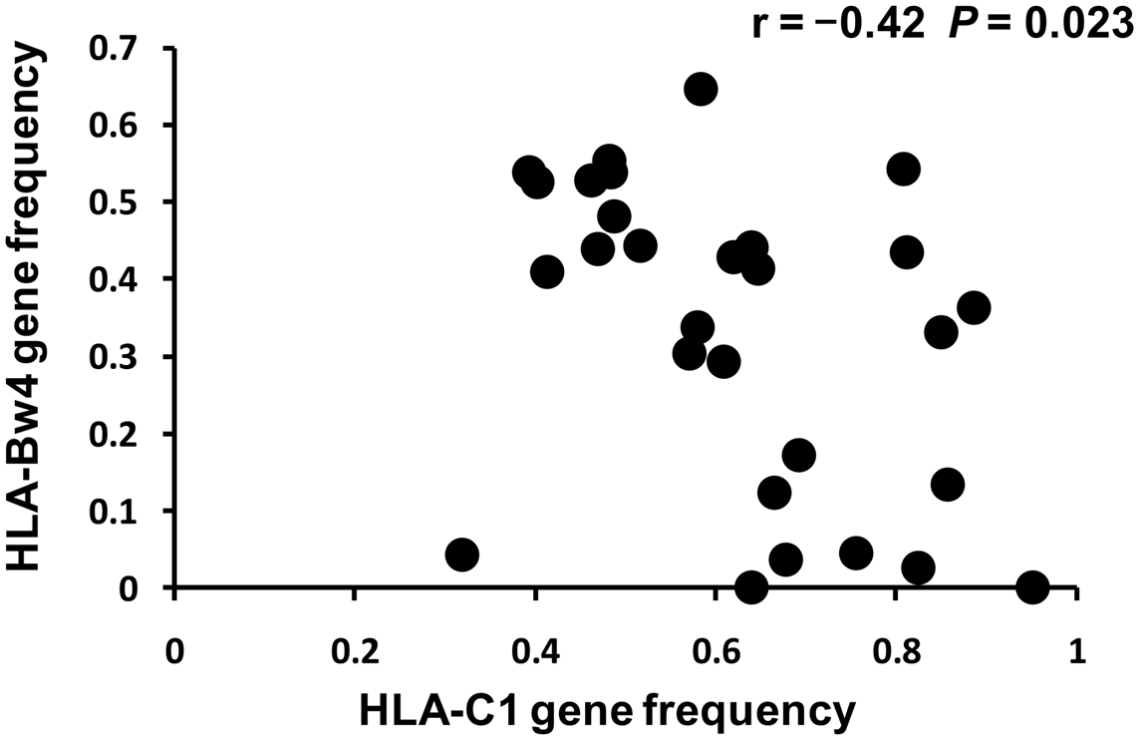
Negative correlation between *HLA-C1* and *HLA-Bw4* gene frequencies in 29 populations. Vertical and horizontal axes indicate *HLA-Bw4* and *HLA-C1* gene frequencies in the 29 populations, respectively. Pearson's product-moment correlation coefficient was applied to these data. Data for the 29 populations were obtained from an earlier report (Single et al., 2007) [15].

The 29 populations were classified into either *P. falciparum* malaria high-endemic or low-endemic populations as described in the methods section, and their GF*GF indices for *KIR2DL3* and *HLA-C1* were compared using the Wilcoxon rank sum test (Figure 3). The GF*GF index was significantly lower in malaria high-endemic than in malaria low-endemic populations (*P* = 0.00045, and supplementary Figure S2). The GF*GF index for the mild malaria group also showed a lower value of 0.52 than that of malaria low-endemic Northeast Asians. In contrast, the GF*GF indices for the combinations of KIR2DL1 with its HLA-C2 ligand and KIR2DL2 with its HLA-C1 ligand showed no significant differences between malaria high-endemic and low-endemic populations. Although the GF*GF index for the combination of KIR3DL1 with HLA-Bw4 was significantly higher in malaria high-endemic than in malaria low-endemic populations (*P* = 0.0042), this is most likely due to a significant negative correlation between HLA-C1 and HLA-Bw4 gene frequencies in these 29 populations (r = −0.42, *P* = 0.023, Figure 4), which is explained by strong linkage disequilibrium between *HLA-C* and *HLA-B* [39].

**Figure 5:**
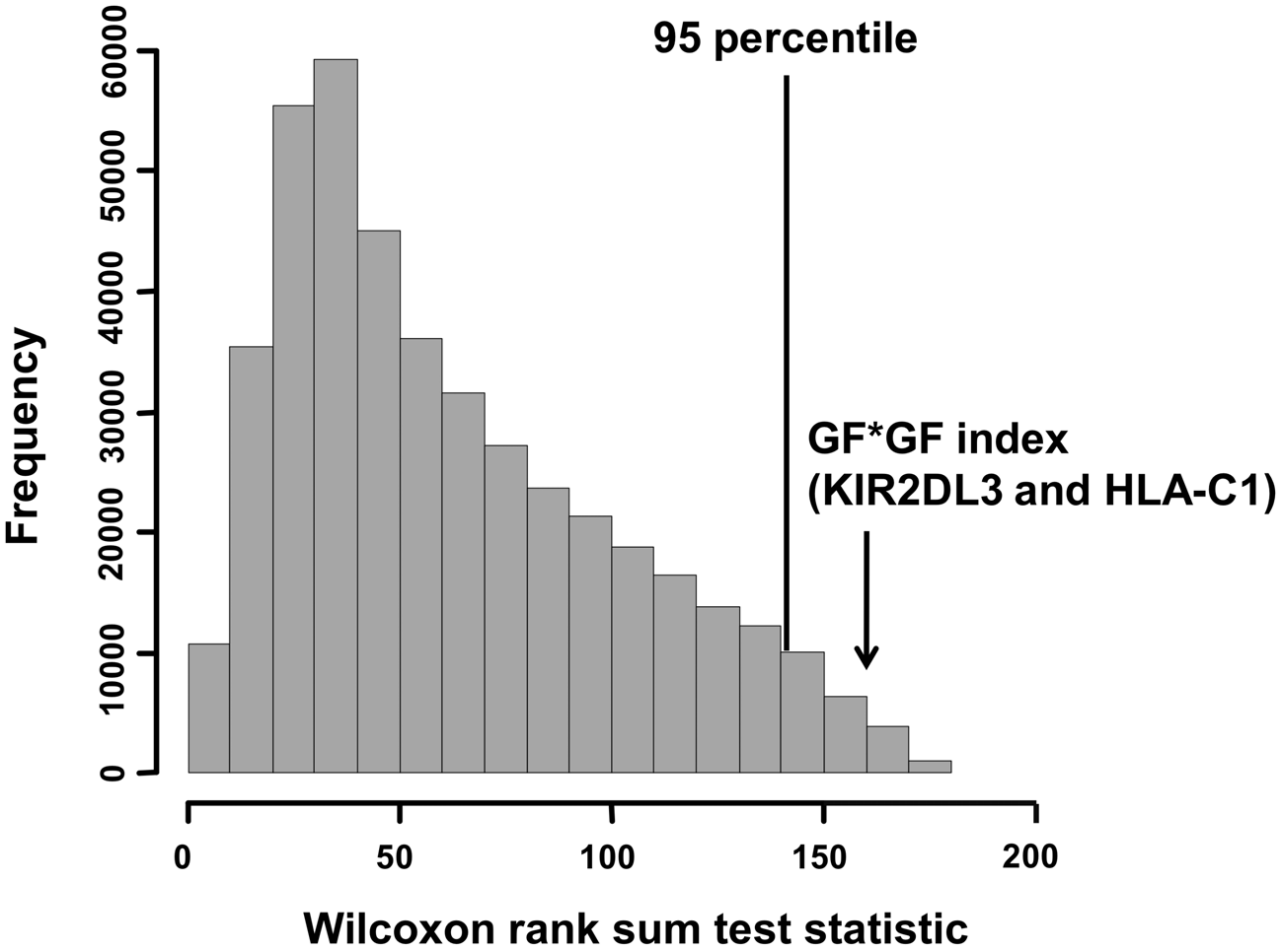
Empirical distribution of the Wilcoxon rank sum test statistics. A total of 429,281 GF*GF indices per population were obtained from 1,051 genome-wide SNPs and compared between malaria high-endemic and low-endemic populations. Horizontal and vertical axes indicate Wilcoxon rank sum test statistics and frequencies, respectively. Data for the 29 populations were obtained from an earlier report (Single et al., 2007) [15].

These results suggest that either or both of the *KIR2DL3* and *HLA-C1* gene frequencies have decreased due to the potentially fatal interaction of KIR2DL3-HLA-C1 in cerebral malaria. In this regard, however, it cannot be ruled out that this observation was confounded by population demographic history of malaria high-endemic and low-endemic regions. Thus, to distinguish between natural selection and the confounding effects of population demographic history, an empirical distribution of the Wilcoxon rank sum test statistics were used to compare the GF*GF indices for 1,051 genome-wide SNPs between malaria high-endemic and low-endemic populations as described in the methods section. On empirical distribution, the GF*GF index of *KIR2DL3* and *HLA-C1* also showed significant difference between malaria high-endemic and low-endemic populations (above the 98^th^ percentile, Figure 5). Taken together, these results suggest that natural selection by cerebral malaria has operated to avoid KIR2DL3-HLA-C1 interaction in malaria high-endemic populations.

## Discussion

Although HLA class I is not expressed on erythrocytes, KIR2DL3-expressing NK cells can respond to inflamed tissues during blood-stage malaria. Artavanis-Tsakonas et al found a significant association between a *KIR3DL2* allele expressed by individual donors and the likelihood of making a strong NK response to *P. falciparum*-infected red blood cells [40]. However, the presence or absence of HLA ligand was unknown, and the *KIR3DL2* allele could not explain all of the NK responses in their study. In addition, NK activation induced by *P. falciparum*-infected red blood cells required myeloid accessory cells [11], [41], interactions described in a “ménage à trois model” [42]. Because myeloid accessory cells express HLA class I, KIR2DL3-expressing NK cells might be able to respond in these conditions. KIR2DL3 binds to HLA-C1 with weaker affinity than does KIR2DL2, an allelic form of KIR2DL3 [20], [43], [44], and therefore HLA-C1-mediated inhibition of NK cells might be weaker in KIR2DL3-expressing NK cells. Alternatively, the association observed in this study might also be explained by a process, described as “licensing”, ”disarming” or “education”, in which the presence of the particular KIR-HLA receptor-ligand pair confers functional competence on NK cells and influences differences in NK cell functional responses among individuals [45]–[50]. Given this observation, the NK cell repertoire shaped by the KIR2DL3-HLA-C1 interaction could exhibit unhelpful responses that increase susceptibility to cerebral malaria. In addition, NK cells were shown to stimulate recruitment of T cells to the brain during *Plasmodium berghei*-mediated cerebral malaria [4]. Thus, NK cells might not directly engage in the pathogenesis of cerebral malaria, but regulate the activation of other immune system cells, which then cause the pathology.

Taniguchi et al reported that *Plasmodium*-positive individuals showed a higher frequency of KIR3DL1/KIR3DS1 heterozygosity than *Plasmodium*-negative individuals [51]. However, KIR3DL1/KIR3DS1 heterozygosity showed no significant association with cerebral malaria in our study (Figure 1). The two studies are different in terms of the outcomes analyzed (infection vs. cerebral malaria), and the *Plasmodium* species analyzed (all four human *Plasmodium* spp. vs. *P. falciparum*). In our study design, which focused on the analysis of cerebral malaria after infection, the effect of the HLA-C1 and KIR2DL3 combination on susceptibility to infection could not be analyzed. To make that possible, it would have been necessary to collect information on the exposure of the subjects to *P. falciparum*.

We previously reported that the allele frequencies of HLA-B46 were statistically different between non-cerebral severe malaria and cerebral malaria using the same cohort [52]. All the individuals carrying HLA-B46 at the HLA-B locus also had HLA-C1 bearing HLA-C owing to the strong linkage disequilibrium in our study population between HLA-B46 and HLA-C1 (D′ = 1), suggesting HLA-B46 as a potential confounder. We also reported that the frequency of some *TNF* alleles were significantly greater in patients with cerebral malaria than in patients with non-cerebral malaria [35]. Although the *TNF* gene is located near *HLA* region, the associated *TNF* alleles were not in linkage disequilibrium with HLA-C1 (D′ = 0.07). The combination of HLA-C1 and KIR2DL3 remained significantly associated with cerebral malaria after adjustment for the HLA-B46 and *TNF* alleles (OR = 2.94, 95%CI 1.47–6.58, *P* = 0.004) by logistic regression analysis, indicating that the combination of HLA-C1 with KIR2DL3 is an independent risk factor for cerebral malaria.

Unexpectedly, the combination of KIR2DS1 and HLA-C2 showed significant association with non-cerebral severe malaria. KIR2DS1 was reported to interact with up-regulated peptide-HLA-C2 complexes on Epstein-Barr virus-infected cells [26]. Therefore, KIR2DS1 might recognize the peptide-HLA-C2 complexes up-regulated by inflammatory conditions during blood-stage malaria.

The Thai patient cohort in this study consisted of residents of Suan Phung. Because other ethnic groups such as Karen and Burmese also resided in this area, we recruited only the patients who were self-identified as Thai. In addition, to exclude a possible spurious association by the population stratification owing to mixture of different ethnic populations, we selected 11 neutral and 7 non-neutral SNPs, which are independent of KIR and HLA, and not in LD with each other, and then, performed association analyses using those SNPs on cerebral and non-cerebral malaria patients. Those analyses suggested that the significant association of KIR2DL3-HLA-C1 combination with cerebral malaria observed in this study is not due to population stratification.

The mean ages for the malaria patients in this study were higher than the mean age of 4.3 years reported for Gambian patients with cerebral malaria [53]. This might be explained by the intensity of malaria transmission. In low and medium transmission settings, cerebral malaria occurs both in adults and children, whereas in high malaria transmission settings, cerebral malaria occurs almost exclusively in infants and young children [54]. The combination of HLA-C1 and KIR2DL3 remained significant even after adjustment for age (OR 3.46, 95%CI 1.63–8.08, *P* = 0.0021).

Malaria was historically common in the Mediterranean littoral, and could therefore have affected the KIR and HLA-C genotype distributions in Europe. However, since malaria endemicity is thought to have been lower in the Mediterranean littoral than in Africa, we classified Europe as “low-endemic region”, assuming that the current relative malaria endemicity is similar to that in the past.

A number of studies have suggested that *KIR* has co-evolved with *HLA* [14], [15], [55], [56]. However, little is known about candidate pathogens acting as strong selective pressures on *KIR* and *HLA*. Our data indicate how selection by malaria could have contributed to the relative frequencies of KIR2DL3 and HLA-C1 in human populations. A recent study showed that natural selection to reduce the frequency and avidity of the KIR2DL3-HLA-C1 interaction has operated in the Yucpa Amerindian tribe living at the border between Venezuela and Colombia [56]. This observation might be partly explained by the selective pressure of malaria, as Colombia is a malaria high-endemic region.

Previous study showed that the KIR2DL3-HLA-C1 conferred a protection against HCV [20]. In contrast, KIR2DL3-HLA-C1 was significantly associated with susceptibility to cerebral malaria in this study. An opposite effect was reported for HIV and HPV, where specific KIR-HLA combinations giving strong NK responses were implicated in resistance to AIDS progression [25] and in susceptibility to HPV-related cervical carcinoma [57]. These observations suggest that a stronger activating KIR-HLA combination is advantageous for clearance of pathogen, but is more likely to cause the severity of disease owing to excessive response.

Taken together, the present results show a significant association between the KIR2DL3-HLA-C1 receptor-ligand pair and cerebral malaria, and the signature of natural selection acting on both *KIR2DL3* and *HLA-C1* due to cerebral malaria. It has been reported that NK cells are required for vaccine-induced protective immunity [58], indicating that understanding the roles of NK cells in malaria is useful for a new vaccine design and a new therapy focused on NK cells. Therefore, our results could have implications for malaria control strategies.

## Materials and Methods

### Ethics Statement

This study was approved by the institutional review board of the Faculty of Tropical Medicine, Mahidol University (Approval reference number: TM-IRB 39), and the Research Ethics Committee of the Graduate School of Comprehensive Human Sciences, University of Tsukuba (Approval reference number: 148-1). Written informed consent was obtained from all patients.

### Patients

A case-control study was conducted of 477 malaria patients (203 mild malaria, 165 non-cerebral severe malaria, and 109 cerebral malaria patients) living in Suan Phung, Ratchaburi-Province, Northwest Thailand. This cohort was designed for the analysis of genetic factors associated with cerebral or severe malaria after infection and DNA samples of these patients were previously collected [59]. We recruited only the patients who were self-identified Thai, and excluded Karen and Burmese. A normal healthy control population from the same area was not included in this study because the primary interest of this study was the development of cerebral malaria after infection. All patients underwent treatment at the Hospital for Tropical Diseases, Faculty of Tropical Medicine, Mahidol University. Clinical manifestations of malaria were classified according to the definitions and associated criteria published by world health organization (WHO) 2000. Cerebral malaria was defined as unrousable coma (Glasgow coma scale of 9 or less), positive blood smear for the asexual form of *P. falciparum* and exclusion of other causes of coma. Non-cerebral severe malaria was defined as having a positive blood smear and fever in addition to one of the following signs: high parasitemia (>100,000 parasites/µL), hypoglycemia (glucose level <2.2 mmol/L), severe anemia (hematocrit <20% or hemoglobin level <7.0 g/dL), and increased serum levels of creatinine (>3.0 mg/dL). Mild malaria was characterized by a positive blood smear and fever without other causes of infections and had no manifestations of severe malaria as described above. Patients aged 13 years or older were analyzed in this study, and the mean ages for patients with mild, non-cerebral severe, and cerebral malaria were 25.5, 23.7 and 28.6 years, respectively. Genomic DNA was extracted from peripheral blood leukocytes using a QIAamp blood kit (Qiagen).

### *HLA* and *KIR* Genotyping

Alleles at the *HLA-A*, *HLA-B* and *-C* loci were determined using a Luminex Multi-Analyte Profiling system (xMAP) with a WAKFlow HLA typing kit (Wakunaga, Hiroshima, Japan), which is based on polymerase chain reaction-reverse sequence-specific oligonucleotide probes (PCR-rSSOP), according to the manufacturer's instructions. The number of probes for *HLA-A*, *HLA-B* and *HLA-C* was 72, 92 and 48, respectively. HLA-Bw4, HLA-C1 and HLA-C2 KIR ligands were assigned based on the amino acid residues of the *HLA-A*, *HLA-B* and *HLA-C* alleles, as described previously [25], [60]. *KIR* genotyping was performed using xMAP with KIR SSO Genotyping Test, lot #002 (One Lambda, Canoga Park, CA), according to the manufacturer's instructions. The presence or absence of the following 16 *KIR* genes was identified: *KIR2DL1*, *KIR2DL2*, *KIR2DL3*, *KIR2DL4*, *KIR2DL5*, *KIR2DS1*, *KIR2DS2*, *KIR2DS3*, *KIR2DS4*, *KIR2DS5*, *KIR3DL1*, *KIR3DL2*, *KIR3DL3*, *KIR3DS1*, *KIR2DP1* and *KIR3DP1*. *KIR2DL5A* and *KIR2DL5B* genes could not be distinguished using this typing system. *KIR* gene profiles were determined by the presence or absence of each *KIR* gene in a given individual. The genotypes of KIR AA or Bx, centromeric (Cen-A/B) or telomeric (Tel-A/B) parts of the KIR genes were deduced from the KIR profiles as defined previously [28], [29], [30]. Cen-B1 and Cen-B2 were grouped together as Cen-B in this study. *KIR* gene profiles are shown in Figure 1. One individual in the non-cerebral severe malaria group was negative for the *KIR2DL4* gene, which is considered to be present in virtually all individuals and is termed a framework *KIR* gene. *KIR* profile #38 of this individual was identical to that of one Bubi individual in an earlier study [61].

### Statistical Analysis

Carrier frequencies for each KIR-HLA receptor-ligand pair were compared between the cerebral malaria patient group and the group of non-cerebral malaria or mild malaria patients using the Fisher's exact test based on a 2×2 contingency table. To further assess the effects of the KIR-HLA receptor-ligand pair of interest on cerebral malaria, a logistic regression analysis was performed after adjustment for age, in which the presence or absence of the specific KIR-HLA receptor-ligand pair, and age (linear) were independent variables. OR and 95%CI were estimated in order to examine the effect size of the association. P values of <0.01 were regarded as statistically significant. Bonferroni correction for multiple testing was applied to our data of KIR-HLA combinations using the number of comparisons performed by our primary factors of interest in Table 1 (i.e. 30 tests = 10 combinations ×3 comparisons between two groups). Bonferroni correction for multiple testing was also applied to the additional analysis of HLA using the additional number of comparisons in Table 2 (i.e. 30 tests +7 additional factors ×3 comparisons between two groups = 51 tests).

In order to exclude the possibility of population stratification, distribution of P values obtained from the SNPs, which are independent of KIR and HLA, was constructed to examine whether P values were not biased toward false positive association. A total of 18 SNPs, some of which were genotyped previously, or additionally genotyped in this study, were used. These 18 SNPs were divided into 11 neutral and 7 non-neutral SNPs, as evaluated by heterozygosity, *F*_ST_ and iHS statistics using Human Evolution Database (http://124.16.129.22/db/table.php) [37]. The procedure was performed as follows; two SNPs were randomly selected from 11 neutral or 7 non-neutral SNPs, resulting in a total of 220 or 84 combinations, respectively, and then, carrier frequencies for one allele in one SNP and one allele in another SNP were compared between cerebral and non-cerebral malaria groups by chi-square test with one degree of freedom. We tested for uniformity of P-value distributions using the chi-square test of goodness-of-fit.

In order to test our hypothesis that the *KIR2DL3* and *HLA-C1* pair, which showed a significant association with cerebral malaria in this study, has been under natural selection in malaria high-endemic regions, we compared the frequency of the KIR2DL3-HLA-C1 combination between malaria high-endemic and low-endemic populations using *KIR2DL3* and *HLA-C1* gene frequencies. The *KIR* carrier frequency (CF) data for 29 populations were obtained from an earlier report [15]. Gene frequencies (GF) of *HLA-C1*, *HLA-C2*, *KIR2DL2*, *KIR2DL3*, and *KIR3DL1* were calculated from the carrier frequencies, assuming that *HLA-C1*, *KIR2DL3*, and *KIR3DL1* are allelic to *HLA-C2*, *KIR2DL2*, and *KIR3DS1*, respectively. Gene frequencies of *HLA-Bw4* and *KIR2DL1* were estimated according to the formula, 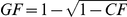, as described previously [15], since information about another allele of the same locus were not available for these two loci.

We used the product of *KIR2DL3* and *HLA-C1* gene frequencies in a population as a measure, which we call the GF*GF index, of the frequency of the KIR2DL3-HLA-C1 combination in that population. The GF*GF index is defined as the product of two different gene frequencies. To compare the frequencies of KIR2DL3-HLA-C1 combination between malaria high-endemic and low-endemic groups, the GF*GF index of *KIR2DL3* and *HLA-C1* was calculated for 29 populations that were classified as either malaria high-endemic or low-endemic groups, and assessed using Wilcoxon rank sum test. Malaria high-endemic areas in this study were defined as areas where more than 25% of malaria cases are due to *P. falciparum* as described in the WHO report [62] (Figure 2). This analysis is based on the relative malaria endemicity, assuming that current relative malaria endemicity is similar to the past.

In order to distinguish between natural selection and the confounding effects of population demographic history, an empirical genome-wide distribution of Wilcoxon rank sum test statistics was constructed using the following procedures. First, a total of 1,051 genome-wide SNPs in 29 populations were obtained from the Allele Frequency Database (ALFRED) [63], a web-based freely accessible compilation of allele frequency data on DNA sequence polymorphisms in anthropologically defined human populations (http://alfred.med.yale.edu). Second, as both *KIR2DL3* and *HLA-C1* are ancestral alleles [64], ancestral allele frequencies were selected from these 1,051 SNPs. The ancestral allele of a SNP was determined by the comparison of human DNA to chimpanzee DNA, and available at dbSNP FTP site (ftp://ftp.ncbi.nih.gov/snp). Third, as both *KIR2DL3* and *HLA-C1* are located on different chromosomes, the GF*GF index of two ancestral alleles on different chromosomes was calculated for each population. Consequently, a total of 429,281 GF*GF indices per population were obtained and compared between malaria high-endemic and low-endemic groups using the Wilcoxon rank sum test. Values beyond the 95^th^ percentile were regarded as significantly lower. To assess the significance of the correlation between *HLA-C1* and *HLA-Bw4* gene frequencies in the 29 populations, Pearson's product-moment correlation coefficient was used.

## Supporting Information

Figure S1 Distributions of p values obtained from association analyses using SNPs independent of *KIR* and *HLA*. Distributions of p values obtained from 11 neutral (A), and 7 non-neutral (B) SNPs corresponding to supplementary Table 3 and supplementary Table 4, respectively, were shown. Neither distribution of p values was biased toward false positive association.
(TIF)

Figure S2 Worldwide frequencies of HLA-C1, KIR2DL3 and combination of HLA-C1 and 2DL3. The location of the pie chart corresponds to the 29 worldwide populations in Figure 2. The frequencies of HLA-C1 (A), KIR2DL3 (B), and combination of HLA-C1 and KIR2DL3 (C) are indicated by red, light blue, and pink, respectively. The frequency of combination of HLA-C1 and KIR2DL3 represents the GF*GF index in Figure 3A. The frequencies of HLA-C1 and KIR2DL3 were obtained from an earlier report (Single et al., 2007) [15].
(TIF)

Table S1 Frequencies of the HLA-C alleles in malaria patient groups and Thai population. There were no significant differences in the frequencies of each individual HLA-C allele between our malaria patients and Thai population that were HLA typed to four digit resolution, and obtained from the Allele Frequency Net Database (population: Thailand) [24].
(DOC)

Table S2 Frequencies of the combinations of KIR2DL3 and each HLA-C1 allele in malaria patient groups. There were no significant differences in the frequencies of the combinations of KIR2DL3 and each HLA-C1 allele (C*01, C*03, C*07, C*08, C*12, and C*14) between malaria patient groups. This observation suggests that the significant association of KIR2DL3-HLA-C1 combination with cerebral malaria does not come from a specific HLA-C1 allele.
(DOC)

Table S3 Detailed information on 11 neutral SNPs used for distribution of p values in supplementary Figure 1A. These 11 SNPs exhibit no LD and are independent of KIR and HLA. For comparison, the HapMap data (JPT+CHB, CEU, and YRI) are shown.
(DOC)

Table S4 Detailed information on 7 non-neutral SNPs used for distribution of p values in supplementary Figure 1B. These 7 SNPs exhibit no LD and are independent of KIR and HLA. These were regarded as non-neutral because one of the three statistics, heterozygosity, *F*_ST_ and iHS, reached statistical significance. For comparison, the HapMap data (JPT+CHB, CEU, and YRI) are shown.
(DOC)

## References

[1] Snow RW, Guerra CA, Noor AM, Myint HY, Hay SI (2005). The global distribution of clinical episodes of Plasmodium falciparum malaria.. Nature.

[2] Marsh K, Forster D, Waruiru C, Mwangi I, Winstanley M (1995). Indicators of life-threatening malaria in African children.. N Engl J Med.

[3] Hansen DS, Siomos MA, Buckingham L, Scalzo AA, Schofield L (2003). Regulation of murine cerebral malaria pathogenesis by CD1d-restricted NKT cells and the natural killer complex.. Immunity.

[4] Hansen DS, Bernard NJ, Nie CQ, Schofield L (2007). NK cells stimulate recruitment of CXCR3+ T cells to the brain during Plasmodium berghei-mediated cerebral malaria.. J Immunol.

[5] Artavanis-Tsakonas K, Riley EM (2002). Innate immune response to malaria: rapid induction of IFN-gamma from human NK cells by live Plasmodium falciparum-infected erythrocytes.. J Immunol.

[6] D'Ombrain MC, Hansen DS, Simpson KM, Schofield L (2007). gammadelta-T cells expressing NK receptors predominate over NK cells and conventional T cells in the innate IFN-gamma response to Plasmodium falciparum malaria.. Eur J Immunol.

[7] Yanez DM, Manning DD, Cooley AJ, Weidanz WP, van der Heyde HC (1996). Participation of lymphocyte subpopulations in the pathogenesis of experimental murine cerebral malaria.. J Immunol.

[8] Amani V, Vigario AM, Belnoue E, Marussig M, Fonseca L (2000). Involvement of IFN-gamma receptor-medicated signaling in pathology and anti-malarial immunity induced by Plasmodium berghei infection.. Eur J Immunol.

[9] Koch O, Awomoyi A, Usen S, Jallow M, Richardson A (2002). IFNGR1 gene promoter polymorphisms and susceptibility to cerebral malaria.. J Infect Dis.

[10] Korbel DS, Newman KC, Almeida CR, Davis DM, Riley EM (2005). Heterogeneous human NK cell responses to Plasmodium falciparum-infected erythrocytes.. J Immunol.

[11] Newman KC, Korbel DS, Hafalla JC, Riley EM (2006). Cross-talk with myeloid accessory cells regulates human natural killer cell interferon-gamma responses to malaria.. PLoS Pathog.

[12] Marsh SG, Parham P, Dupont B, Geraghty DE, Trowsdale J (2003). Killer-cell immunoglobulin-like receptor (KIR) nomenclature report, 2002.. Tissue Antigens.

[13] Yawata M, Yawata N, Abi-Rached L, Parham P (2002). Variation within the human killer cell immunoglobulin-like receptor (KIR) gene family.. Crit Rev Immunol.

[14] Norman PJ, Abi-Rached L, Gendzekhadze K, Korbel D, Gleimer M (2007). Unusual selection on the KIR3DL1/S1 natural killer cell receptor in Africans.. Nat Genet.

[15] Single RM, Martin MP, Gao X, Meyer D, Yeager M (2007). Global diversity and evidence for coevolution of KIR and HLA.. Nat Genet.

[16] Mandelboim O, Reyburn HT, Vales-Gomez M, Pazmany L, Colonna M (1996). Protection from lysis by natural killer cells of group 1 and 2 specificity is mediated by residue 80 in human histocompatibility leukocyte antigen C alleles and also occurs with empty major histocompatibility complex molecules.. J Exp Med.

[17] Barber LD, Percival L, Valiante NM, Chen L, Lee C (1996). The inter-locus recombinant HLA-B*4601 has high selectivity in peptide binding and functions characteristic of HLA-C.. J Exp Med.

[18] Cella M, Longo A, Ferrara GB, Strominger JL, Colonna M (1994). Nk3-Specific Natural-Killer-Cells Are Selectively Inhibited by Bw4-Positive Hla Alleles with Isoleucine-80.. J Exp Med.

[19] Gumperz JE, Litwin V, Phillips JH, Lanier LL, Parham P (1995). The Bw4 public epitope of HLA-B molecules confers reactivity with natural killer cell clones that express NKB1, a putative HLA receptor.. J Exp Med.

[20] Khakoo SI, Thio CL, Martin MP, Brooks CR, Gao X (2004). HLA and NK cell inhibitory receptor genes in resolving hepatitis C virus infection.. Science.

[21] Miyashita R, Tsuchiya N, Yabe T, Kobayashi S, Hashimoto H (2006). Association of killer cell immunoglobulin-like receptor genotypes with microscopic polyangiitis.. Arthritis Rheum.

[22] Mogami S, Hasegawa G, Nakayama I, Asano M, Hosoda H (2007). Killer cell immunoglobulin-like receptor genotypes in Japanese patients with type 1 diabetes.. Tissue Antigens.

[23] Martin MP, Qi Y, Gao X, Yamada E, Martin JN (2007). Innate partnership of HLA-B and KIR3DL1 subtypes against HIV-1.. Nat Genet.

[24] Gonzalez-Galarza FF, Christmas S, Middleton D, Jones AR (2011). Allele frequency net: a database and online repository for immune gene frequencies in worldwide populations.. Nucleic Acids Res.

[25] Martin MP, Gao X, Lee JH, Nelson GW, Detels R (2002). Epistatic interaction between KIR3DS1 and HLA-B delays the progression to AIDS.. Nat Genet.

[26] Stewart CA, Laugier-Anfossi F, Vely F, Saulquin X, Riedmuller J (2005). Recognition of peptide-MHC class I complexes by activating killer immunoglobulin-like receptors.. Proc Natl Acad Sci U S A.

[27] Graef T, Moesta AK, Norman PJ, Abi-Rached L, Vago L (2009). KIR2DS4 is a product of gene conversion with KIR3DL2 that introduced specificity for HLA-A*11 while diminishing avidity for HLA-C.. J Exp Med.

[28] Pyo CW, Guethlein LA, Vu Q, Wang R, Abi-Rached L (2010). Different patterns of evolution in the centromeric and telomeric regions of group A and B haplotypes of the human killer cell Ig-like receptor locus.. PLoS One.

[29] Hou L, Chen M, Ng J, Hurley CK (2012). Conserved KIR allele-level haplotypes are altered by microvariation in individuals with European ancestry.. Genes Immun.

[30] Cooley S, Weisdorf DJ, Guethlein LA, Klein JP, Wang T (2010). Donor selection for natural killer cell receptor genes leads to superior survival after unrelated transplantation for acute myelogenous leukemia.. Blood.

[31] Ohashi J, Naka I, Patarapotikul J, Hananantachai H, Looareesuwan S (2001). Absence of association between the allele coding methionine at position 29 in the N-terminal domain of ICAM-1 (ICAM-1(Kilifi)) and severe malaria in the northwest of Thailand.. Jpn J Infect Dis.

[32] Omi K, Ohashi J, Patarapotikul J, Hananantachai H, Naka I (2002). Fcgamma receptor IIA and IIIB polymorphisms are associated with susceptibility to cerebral malaria.. Parasitol Int.

[33] Ohashi J, Naka I, Patarapotikul J, Hananantachai H, Looareesuwan S (2002). Lack of association between interleukin-10 gene promoter polymorphism, -1082G/A, and severe malaria in Thailand.. Southeast Asian J Trop Med Public Health.

[34] Ohashi J, Naka I, Patarapotikul J, Hananantachai H, Looareesuwan S (2003). A single-nucleotide substitution from C to T at position -1055 in the IL-13 promoter is associated with protection from severe malaria in Thailand.. Genes Immun.

[35] Hananantachai H, Patarapotikul J, Ohashi J, Naka I, Krudsood S (2007). Significant association between TNF-alpha (TNF) promoter allele (-1031C, -863C, and -857C) and cerebral malaria in Thailand.. Tissue Antigens.

[36] Teeranaipong P, Ohashi J, Patarapotikul J, Kimura R, Nuchnoi P (2008). A functional single-nucleotide polymorphism in the CR1 promoter region contributes to protection against cerebral malaria.. J Infect Dis.

[37] Cheng F, Chen W, Richards E, Deng L, Zeng C (2009). SNP@Evolution: a hierarchical database of positive selection on the human genome.. BMC Evol Biol.

[38] Cooke GS, Hill AV (2001). Genetics of susceptibility to human infectious disease.. Nat Rev Genet.

[39] de Bakker PI, McVean G, Sabeti PC, Miretti MM, Green T (2006). A high-resolution HLA and SNP haplotype map for disease association studies in the extended human MHC.. Nat Genet.

[40] Artavanis-Tsakonas K, Eleme K, McQueen KL, Cheng NW, Parham P (2003). Activation of a subset of human NK cells upon contact with Plasmodium falciparum-infected erythrocytes.. J Immunol.

[41] Baratin M, Roetynck S, Lepolard C, Falk C, Sawadogo S (2005). Natural killer cell and macrophage cooperation in MyD88-dependent innate responses to Plasmodium falciparum.. Proc Natl Acad Sci U S A.

[42] Roetynck S, Baratin M, Johansson S, Lemmers C, Vivier E (2006). Natural killer cells and malaria.. Immunol Rev.

[43] Winter CC, Gumperz JE, Parham P, Long EO, Wagtmann N (1998). Direct binding and functional transfer of NK cell inhibitory receptors reveal novel patterns of HLA-C allotype recognition.. J Immunol.

[44] Moesta AK, Norman PJ, Yawata M, Yawata N, Gleimer M (2008). Synergistic polymorphism at two positions distal to the ligand-binding site makes KIR2DL2 a stronger receptor for HLA-C than KIR2DL3.. J Immunol.

[45] Kim S, Poursine-Laurent J, Truscott SM, Lybarger L, Song YJ (2005). Licensing of natural killer cells by host major histocompatibility complex class I molecules.. Nature.

[46] Fernandez NC, Treiner E, Vance RE, Jamieson AM, Lemieux S (2005). A subset of natural killer cells achieves self-tolerance without expressing inhibitory receptors specific for self-MHC molecules.. Blood.

[47] Anfossi N, Andre P, Guia S, Falk CS, Roetynck S (2006). Human NK cell education by inhibitory receptors for MHC class I.. Immunity.

[48] Yu J, Heller G, Chewning J, Kim S, Yokoyama WM (2007). Hierarchy of the human natural killer cell response is determined by class and quantity of inhibitory receptors for self-HLA-B and HLA-C ligands.. J Immunol.

[49] Kim S, Sunwoo JB, Yang L, Choi T, Song YJ (2008). HLA alleles determine differences in human natural killer cell responsiveness and potency.. Proc Natl Acad Sci U S A.

[50] Yawata M, Yawata N, Draghi M, Partheniou F, Little AM (2008). MHC class I-specific inhibitory receptors and their ligands structure diverse human NK-cell repertoires toward a balance of missing self-response.. Blood.

[51] Taniguchi M, Kawabata M (2009). KIR3DL1/S1 genotypes and KIR2DS4 allelic variants in the AB KIR genotypes are associated with Plasmodium-positive individuals in malaria infection.. Immunogenetics.

[52] Hananantachai H, Patarapotikul J, Ohashi J, Naka I, Looareesuwan S (2005). Polymorphisms of the HLA-B and HLA-DRB1 genes in Thai malaria patients.. Jpn J Infect Dis.

[53] Jallow M, Teo YY, Small KS, Rockett KA, Deloukas P (2009). Genome-wide and fine-resolution association analysis of malaria in West Africa.. Nat Genet.

[54] White NJ (2004). Cerebral malaria.. Pract Neurol.

[55] Hiby SE, Walker JJ, O'Shaughnessy KM, Redman CW, Carrington M (2004). Combinations of maternal KIR and fetal HLA-C genes influence the risk of preeclampsia and reproductive success.. J Exp Med.

[56] Gendzekhadze K, Norman PJ, Abi-Rached L, Graef T, Moesta AK (2009). Co-evolution of KIR2DL3 with HLA-C in a human population retaining minimal essential diversity of KIR and HLA class I ligands.. Proc Natl Acad Sci U S A.

[57] Carrington M, Wang S, Martin MP, Gao X, Schiffman M (2005). Hierarchy of resistance to cervical neoplasia mediated by combinations of killer immunoglobulin-like receptor and human leukocyte antigen loci.. J Exp Med.

[58] Doolan DL, Hoffman SL (1999). IL-12 and NK cells are required for antigen-specific adaptive immunity against malaria initiated by CD8+ T cells in the Plasmodium yoelii model.. J Immunol.

[59] Ohashi J, Naka I, Patarapotikul J, Hananantachai H, Looareesuwan S (2002). Significant association of longer forms of CCTTT Microsatellite repeat in the inducible nitric oxide synthase promoter with severe malaria in Thailand.. J Infect Dis.

[60] Yabe T, Matsuo K, Hirayasu K, Kashiwase K, Kawamura-Ishii S (2008). Donor killer immunoglobulin-like receptor (KIR) genotype-patient cognate KIR ligand combination and antithymocyte globulin preadministration are critical factors in outcome of HLA-C-KIR ligand-mismatched T cell-replete unrelated bone marrow transplantation.. Biol Blood Marrow Transplant.

[61] Gomez-Lozano N, de Pablo R, Puente S, Vilches C (2003). Recognition of HLA-G by the NK cell receptor KIR2DL4 is not essential for human reproduction.. Eur J Immunol.

[62] WHO (2008). World Health Organization World malaria report 2008.. http://apps.who.int/malaria/.

[63] Rajeevan H, Osier MV, Cheung KH, Deng H, Druskin L (2003). ALFRED: the ALelle FREquency Database. Update.. Nucleic Acids Res.

[64] Moesta AK, Abi-Rached L, Norman PJ, Parham P (2009). Chimpanzees use more varied receptors and ligands than humans for inhibitory killer cell Ig-like receptor recognition of the MHC-C1 and MHC-C2 epitopes.. J Immunol.

