# Supplementary figures and images for: Significant Association of KIR2DL3-HLA-C1 Combination with Cerebral Malaria and Implications for Co-evolution of KIR and HLA
Source: PLoS Pathog. 2012 Mar 8;8(3):e1002565. doi: 10.1371/journal.ppat.1002565 (PMC3297587; doi:10.1371/journal.ppat.1002565)

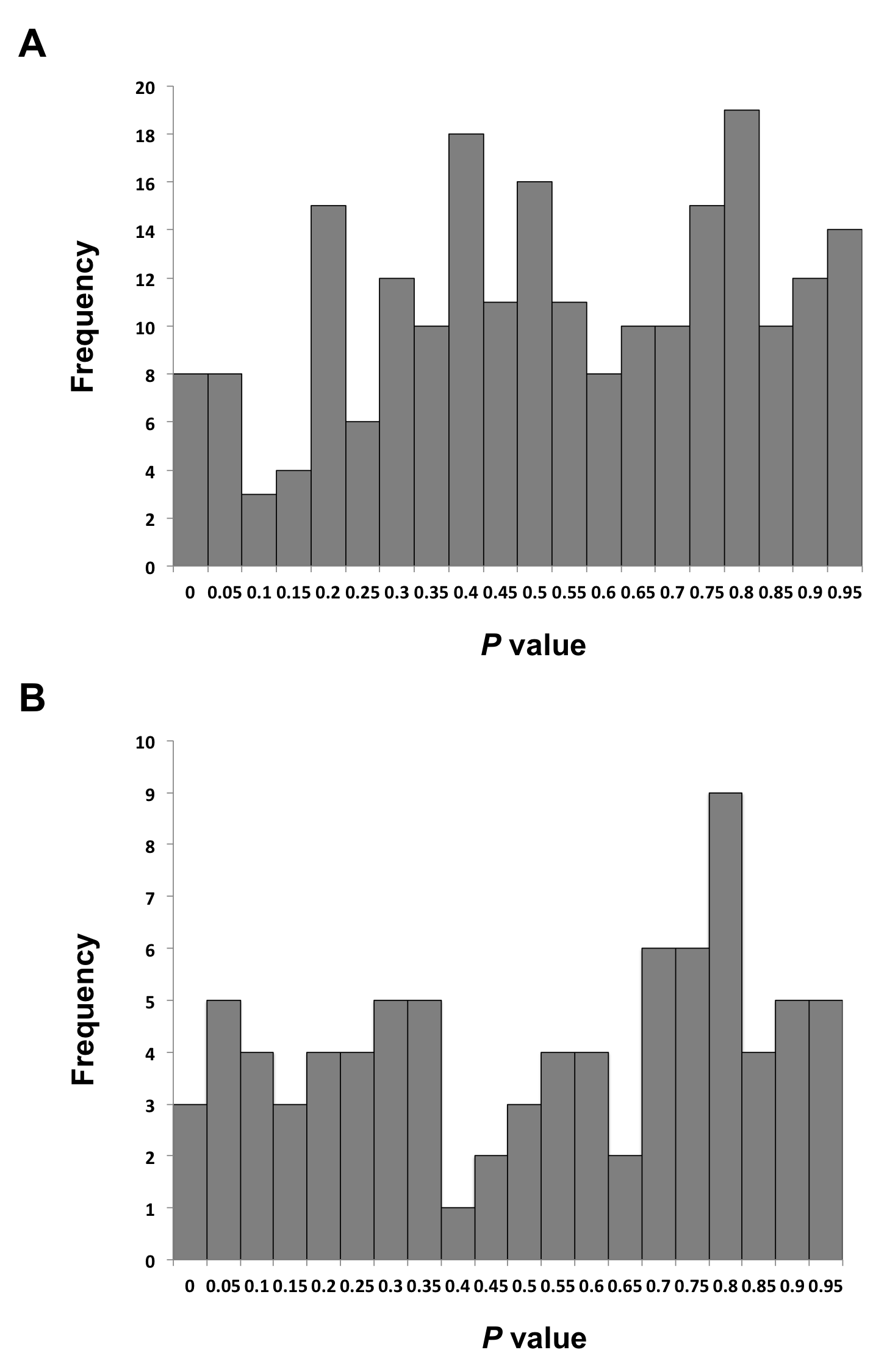

Supplement: Figure S1 — Distributions of p values obtained from association analyses using SNPs independent of KIR and HLA. Distributions of p values obtained from 11 neutral (A), and 7 non-neutral (B) SNPs corresponding to supplementary Table 3 and supplementary Table 4, respectively, were shown. Neither distribution of p values was biased toward false positive association. (TIF) [file ppat.1002565.s001.tif]

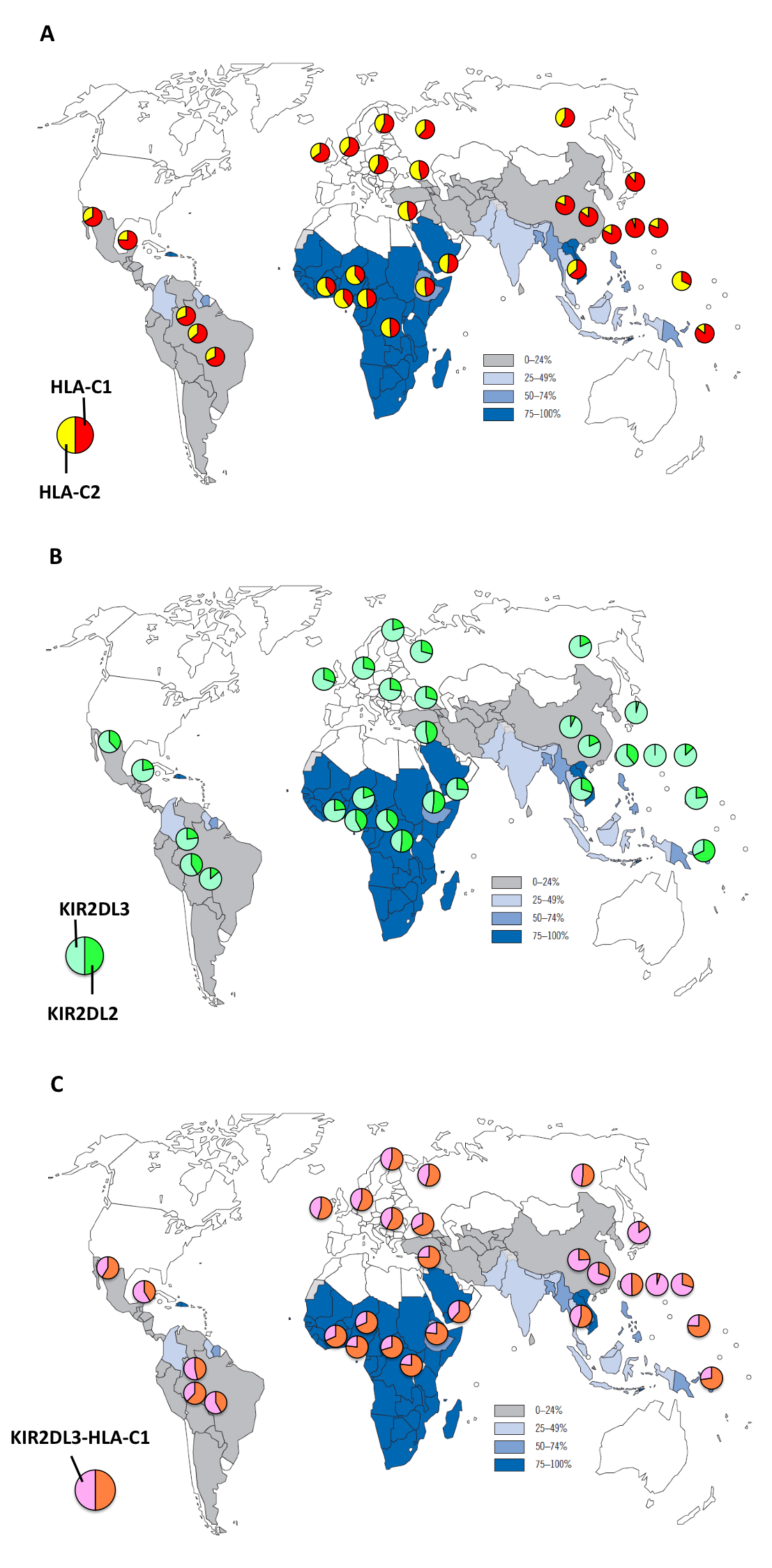

Supplement: Figure S2 — Worldwide frequencies of HLA-C1, KIR2DL3 and combination of HLA-C1 and 2DL3. The location of the pie chart corresponds to the 29 worldwide populations in Figure 2. The frequencies of HLA-C1 (A), KIR2DL3 (B), and combination of HLA-C1 and KIR2DL3 (C) are indicated by red, light blue, and pink, respectively. The frequency of combination of HLA-C1 and KIR2DL3 represents the GF*GF index in Figure 3A. The frequencies of HLA-C1 and KIR2DL3 were obtained from an earlier report (Single et al., 2007) [15]. (TIF) [file ppat.1002565.s002.tif]
