# Supplementary material for: Significant Association of KIR2DL3-HLA-C1 Combination with Cerebral Malaria and Implications for Co-evolution of KIR and HLA
Source: PLoS Pathog. 2012 Mar 8;8(3):e1002565. doi: 10.1371/journal.ppat.1002565 (PMC3297587; doi:10.1371/journal.ppat.1002565)
Supplement: Table S1 — Frequencies of the HLA-C alleles in malaria patient groups and Thai population. There were no significant differences in the frequencies of each individual HLA-C allele between our malaria patients and Thai population that were HLA typed to four digit resolution, and obtained from the Allele Frequency Net Database (population: Thailand) [24]. (DOC) [file ppat.1002565.s003.doc]

Supplementary Table 1 Frequencies of the *HLA-C* alleles in malaria patient groups and Thai population

| HLA-C | C1/C2 | Cerebral (2n=218) | Non-cerebral severe (2n=330) | Mild (2n=406) | Thailand^a^ (2n=284) |
| --- | --- | --- | --- | --- | --- |
| C*01:02 | C1 | 0.142 | 0.106 | 0.128 | 0.116 |
| C*02:02 | C2 | 0.000 | 0.003 | 0.000 | 0.004 |
| C*03:02 | C1 | 0.073 | 0.058 | 0.042 | 0.081 |
| C*03:03 | C1 | 0.014 | 0.024 | 0.012 | 0.018 |
| C*03:04 | C1 | 0.055 | 0.061 | 0.049 | 0.095 |
| C*04:01 | C2 | 0.055 | 0.076 | 0.054 | 0.060 |
| C*04:03 | C2 | 0.050 | 0.088 | 0.067 | 0.053 |
| C*04:06 | C2 | 0.018 | 0.012 | 0.017 | 0.000 |
| C*05:01 | C2 | 0.000 | 0.000 | 0.002 | 0.000 |
| C*06:02 | C2 | 0.037 | 0.055 | 0.059 | 0.046 |
| C*07:01 | C1 | 0.096 | 0.082 | 0.081 | 0.077 |
| C*07:02 | C1 | 0.193 | 0.142 | 0.160 | 0.130 |
| C*07:04 | C1 | 0.009 | 0.024 | 0.032 | 0.042 |
| C*08:01 | C1 | 0.119 | 0.121 | 0.150 | 0.120 |
| C*08:02 | C1 | 0.000 | 0.000 | 0.002 | 0.000 |
| C*12:02 | C1 | 0.046 | 0.036 | 0.039 | 0.046 |
| C*12:03 | C1 | 0.032 | 0.030 | 0.032 | 0.014 |
| C*12:04 | C2 | 0.000 | 0.006 | 0.000 | 0.000 |
| C*14:02 | C1 | 0.018 | 0.015 | 0.012 | 0.046 |
| C*15:02 | C2 | 0.037 | 0.055 | 0.049 | 0.046 |
| C*15:04 | C2 | 0.000 | 0.003 | 0.000 | 0.000 |
| C*15:05 | C2 | 0.005 | 0.003 | 0.005 | 0.007 |
| C*16:02 | C2 | 0.000 | 0.000 | 0.005 | 0.000 |

^a^ HLA-C allele frequencies in Thailand are available from the Allele Frequency Net Database (population: Thailand)
