# Supplementary material for: Significant Association of KIR2DL3-HLA-C1 Combination with Cerebral Malaria and Implications for Co-evolution of KIR and HLA
Source: PLoS Pathog. 2012 Mar 8;8(3):e1002565. doi: 10.1371/journal.ppat.1002565 (PMC3297587; doi:10.1371/journal.ppat.1002565)
Supplement: Table S2 — Frequencies of the combinations of KIR2DL3 and each HLA-C1 allele in malaria patient groups. There were no significant differences in the frequencies of the combinations of KIR2DL3 and each HLA-C1 allele (C*01, C*03, C*07, C*08, C*12, and C*14) between malaria patient groups. This observation suggests that the significant association of KIR2DL3-HLA-C1 combination with cerebral malaria does not come from a specific HLA-C1 allele. (DOC) [file ppat.1002565.s004.doc]

Supplementary Table 2 Frequencies of the combinations of KIR2DL3 and each HLA-C1 allele in malaria patient groups

| KIR2DL3+HLA-C | Cerebral (N=109) | Non-cerebral severe (N=165) | Mild (N=203) |
| --- | --- | --- | --- |
| 2DL3+HLA-C*01 | 0.266 | 0.158 | 0.207 |
| 2DL3+HLA-C*03 | 0.239 | 0.242 | 0.177 |
| 2DL3+HLA-C*07 | 0.495 | 0.364 | 0.409 |
| 2DL3+HLA-C*08 | 0.229 | 0.200 | 0.281 |
| 2DL3+HLA-C*12^a^ | 0.147 | 0.103 | 0.123 |
| 2DL3+HLA-C*14 | 0.037 | 0.030 | 0.020 |

^a^ C*12:04 (HLA-C2) was removed from the analysis
