# Supplementary material for: Significant Association of KIR2DL3-HLA-C1 Combination with Cerebral Malaria and Implications for Co-evolution of KIR and HLA
Source: PLoS Pathog. 2012 Mar 8;8(3):e1002565. doi: 10.1371/journal.ppat.1002565 (PMC3297587; doi:10.1371/journal.ppat.1002565)
Supplement: Table S3 — Detailed information on 11 neutral SNPs used for distribution of p values in supplementary Figure 1A. These 11 SNPs exhibit no LD and are independent of KIR and HLA. For comparison, the HapMap data (JPT+CHB, CEU, and YRI) are shown. (DOC) [file ppat.1002565.s005.doc]

Supplementary Table 3 Detailed information on 11 neutral SNPs used for distribution of p values in supplementary Figure 1A

|  |  |  |  | Minor allele frequency | | | | |
| --- | --- | --- | --- | --- | --- | --- | --- | --- |
| rs# (minor allele) | chromosome | location | Flanking gene | Cerebral | Non-cerebral | JPT+CHB | CEU | YRI |
| rs1800896 (G) | 1 | 205013520 | IL10 | 0.073 | 0.076 | 0.049 | 0.531 | 0.274 |
| rs1801274 (C) | 1 | 161479745 | FCGR2A | 0.220 | 0.257 | 0.252 | 0.508 | 0.517 |
| rs352139 (G) | 3 | 52258372 | TLR9 | 0.373 | 0.419 | 0.477 | 0.500 | 0.559 |
| rs7744 (G) | 3 | 38184021 | MYD88 | 0.282 | 0.276 | 0.327 | 0.142 | 0.025 |
| rs1898830 (G) | 4 | 154608453 | TLR2 | 0.349 | 0.415 | 0.410 | 0.350 | 0.067 |
| rs3775291 (A) | 4 | 187004074 | TLR3 | 0.343 | 0.371 | 0.270 | 0.325 | 0.000 |
| rs31480 (T) | 5 | 131396332 | IL3 | 0.389 | 0.396 | 0.561 | 0.198 | 0.136 |
| rs757537 (C) | 5 | 132151071 | ANKRD43 | 0.153 | 0.146 | 0.096 | 0.172 | 0.058 |
| rs2070722 (G) | 5 | 131824486 | IRF1 | 0.389 | 0.414 | 0.306 | 0.392 | 0.550 |
| rs1927914 (T) | 9 | 120464725 | TLR4 | 0.319 | 0.301 | 0.646 | 0.692 | 0.167 |
| rs5491 (T) | 19 | 10385540 | ICAM1 | 0.023 | 0.022 | 0.067 | 0.000 | 0.250 |
