# Supplementary material for: Significant Association of KIR2DL3-HLA-C1 Combination with Cerebral Malaria and Implications for Co-evolution of KIR and HLA
Source: PLoS Pathog. 2012 Mar 8;8(3):e1002565. doi: 10.1371/journal.ppat.1002565 (PMC3297587; doi:10.1371/journal.ppat.1002565)
Supplement: Table S4 — Detailed information on 7 non-neutral SNPs used for distribution of p values in supplementary Figure 1B. These 7 SNPs exhibit no LD and are independent of KIR and HLA. These were regarded as non-neutral because one of the three statistics, heterozygosity, F ST and iHS, reached statistical significance. For comparison, the HapMap data (JPT+CHB, CEU, and YRI) are shown. (DOC) [file ppat.1002565.s006.doc]

Supplementary Table 4 Detailed information on 7 non-neutral SNPs used for distribution of p values in supplementary Figure 1B

|  |  |  |  | Minor allele frequency | | | | |
| --- | --- | --- | --- | --- | --- | --- | --- | --- |
| rs# (minor allele) | chromosome | location | Flanking gene | Cerebral | Non-cerebral | JPT+CHB | CEU | YRI |
| rs1061622 (G) | 1 | 12175542 | TNFR2 | 0.134 | 0.137 | 0.186 | 0.239 | 0.170 |
| rs6691117 (G) | 1 | 205849554 | CR1 | 0.463 | 0.488 | 0.204 | 0.179 | 0.904 |
| rs4833095 (T) | 4 | 38476105 | TLR1 | 0.472 | 0.425 | 0.312 | 0.788 | 0.116 |
| rs5743808 (G) | 4 | 38507131 | TLR6 | 0.127 | 0.134 | 0.057 | n/a | 0.085 |
| rs7657186 (A) | 4 | 187231033 | TLR3 | 0.225 | 0.176 | 0.088 | 0.155 | 0.272 |
| rs2301713 (C) | 5 | 131979895 | RAD50 homolog | 0.159 | 0.157 | 0.193 | 0.204 | 0.340 |
| rs1800629 (A) | 6 | 31651010 | TNF | 0.069 | 0.052 | 0.053 | 0.173 | 0.095 |
